# A prospective study of patients with low back pain attending a Canadian emergency department: Why they came and what happened?

**DOI:** 10.1371/journal.pone.0268123

**Published:** 2022-05-10

**Authors:** Gregory N. Kawchuk, Jacob Aaskov, Matthew Mohler, Justin Lowes, Maureen Kruhlak, Stephanie Couperthwaite, Esther H. Yang, Cristina Villa-Roel, Brian H. Rowe

**Affiliations:** 1 Department of Physical Therapy, Faculty of Rehabilitation Medicine, University of Alberta, Edmonton, Canada; 2 Department of Sports Science and Clinical Biomechanics, University of Southern Denmark, Odense, Denmark; 3 Department of Emergency Medicine, Faculty of Medicine & Dentistry, University of Alberta, Edmonton, Canada; 4 School of Public Health, University of Alberta, Edmonton, Canada; Mugla Sitki Kocman Universitesi, TURKEY

## Abstract

Low back pain is a common presentation to emergency departments, but the reasons why people choose to attend the emergency department have not been explored. We aimed to fill this gap with this study to understand why persons with low back pain choose to attend the emergency department. Between July 4, 2017 and October 1, 2018, consecutive patients with a complaint of low back pain presenting to the University of Alberta Hospital emergency department were screened. Those enrolled completed a 13-item questionnaire to assess reasons and expectations related to their presentation. Demographics, acuity and disposition were obtained electronically. Factors associated with admission were examined in a logistic regression model. After screening 812 patients, 209 participants met the study criteria. The most common Canadian Triage and Acuity Scale score was 3 (73.2%). Overall, 37 (17.7%) received at least one consultation, 89.0% of participants were discharged home, 9.6% were admitted and 1.4% were transferred. Participants had a median pain intensity of 8/10 and a median daily functioning of 3/10. When asked, 64.6% attended for pain control while 44.5% stated ease of access. Most participants expected to obtain pain medication (67%) and advice (56%). Few attended because of cost savings (3.8%). After adjustment, only advanced age and ambulance arrival were significantly associated with admission. In conclusion, most low back pain patients came to the emergency department for pain control yet few were admitted and the majority did not receive a consultation. Timely alternatives for management of low back pain in the emergency department appear needed, yet are lacking.

## Introduction

### Background

Low back pain (LBP), a leading cause of disability in Canada [1], affects over 540 million people globally. With that impact and distribution, there is no shortage of settings in which LBP is evaluated whether in rehabilitation practices, primary care practice or hospitals (out-patient and in-patient settings). Indeed, LBP is a very common presentation to emergency departments (EDs). A recent systematic review by Edwards et al. reported that “LBP is consistently a top presenting complaint” within EDs [2]. In Canada, a multi-year analysis [3] showed the prevalence of LBP in a Canadian ED to be approximately 3% and the sixth most common reason for acute presentations [4]. From these studies, a considerable amount of LBP ED presentation characteristics is known including patient demographics, triage scores, length of stay (LOS), use of resources (e.g., imaging, consultations) and outcomes. This includes recent knowledge that the common practice of admitting patients with non-serious low back pain for inpatient care comes at great expense to the healthcare system [5] while many people admitted to hospital with a provisional diagnosis of nonserious back pain are subsequently found to have serious pathology as the underlying cause [6].

### Importance

Despite this information, little is known about why people with LBP choose to come to the ED when other options are available in the community. In counties with universal publicly–funded health care like Canada, these options include no-cost access to primary care providers in the community and access to fee-for-service allied health care professions (e.g. chiroprators, physical therapists). This is an important question as very few patients with LBP who present to the ED are admitted to hospital. In many hospital settings, less than 10% of LBP cases presenting to the ED are admitted [3]. These low admittance rates suggest that the majority of ED LBP cases could be managed effectively by primary care providers; an important point as evaluating LBP in the ED can be associated with a higher frequency of imaging and opioid prescription [7–9] as Canadian EDs lacks national guidelines regarding LBP evaluation [10].

### Goals of this investigation

Given the above, the objective of this study was to document the reasons that persons with LBP present to the ED as they pertain to accessibility, social and economic domains. In addition, factors associated with admission were examined in an effort to generate future hypotheses. To our knowledge, the answers to these questions have not been reported previously in the emergency literature.

## Materials and methods

### Study design and setting

This was a prospective observational, monocentric study whose target population was those presenting to the University of Alberta Hospital Emergency Department (UAHED) with a complaint of LBP. The UAHED is a major urban teaching center located in Edmonton, Alberta, Canada (population = 1.3 million {2017}), with approximately 75,000 adult patient visits per year. It is a trauma, burns, transplant and pediatric centre staffed by full time emergency physicians and learners from a variety of specialty programs. A total of 11 clinical shifts of eight hours duration are employed to staff the adult side of the UAHED. At the time of the study, no other health care professionals (e.g., nurse practitioners, paramedics, physicians’ assistants, etc.) were employed at this site. At this institution, the on-call spine service alternates daily between Orthopedic and Neurosurgical services.

### Selection of participants

Included participants were persons aged 18 years of age or older visiting the UAHED with who self-reported LBP. Those who were deemed competent to provide informed consent were eligible for the study. At triage, Canadian Triage and Acuity Scale (CTAS) scores [11] were assigned by an experienced triage nurse. The CTAS includes a five-level triage scoring system: Resuscitation (CTAS 1), Emergent (CTAS 2), Urgent (CTAS 3), Less Urgent (CTAS 4), and Non-Urgent (CTAS 5).

Those excluded from the study were considered to have cognitive impairment, were enrolled previously in the study, attended for direct consultations, or were patients presenting to UAHED under police escort. Further, patients who were unable to read or communicate in English were also excluded, unless a friend or family member was able to assist in the completion of the collection of study materials. Patients who were feeling too unwell, due to nausea, pain, emotional instability, or intoxication, but improved before the end of the study recruitment shift, were approached to participate in the study. A Refused, Missed, and Other exclusion (RMO) minimal data log was maintained (e.g., age, sex, time of day, triage score, reason for exclusion). In addition to those patients approached directly by the research assistants, a sign was approved and posted in the ED informing those in the waiting room about the study. Interested patients followed the informed consent process detailed above.

The study protocol was approved by to the Biomedical Panel of the Health Research Ethics Board (HREB) at the University of Alberta (Pro00049637). Operational/administrative approval was provided by Alberta Health Services (AHS) to permit data collection at the study site.

### Measurements

The study questionnaire was developed from a prior pilot mailout survey with a subsequent response rate of ~ 15% [11]. Based on this low response rate, we modified the mailout survey to be used as an onsite questionnaire in the ED at the time of patient presentation. The final questions used in this questionnaire were a subset from the prior mailout survey and selected by the expert panel of collaborators as those related directly to the main research question. The questionnaire had face and content validity and was pilot tested prior to use; however, this is the first use of the questionnaire and no formal validation study was conducted. Questionnaire access was closed in that it was made available only to those persons who provided consent and met the enrollment criteria.

On most occasions, the research assistant approached and interviewed the patient prior to an emergency physician seeing the patient. During the interview, if a physician or nurse needed to assess the patient, the interview was completed after their assessment. Participation in the study was voluntary, no incentives were offered to patients for participation, and completion did not delay direct patient care. The questionnaire was taken only by those who provided consent and in no way affected their care.

Enrollment commenced on July 14, 2017 and ended June 30, 2018.

All responses were collected on paper forms and checked for completeness by our research staff. All data were then entered into a password protected Research Electronic Data Capture (REDCap) website (Vanderbilt University license to the Women and Children’s Health Research Institute—WCHRI, University of Alberta) located on a secure University of Alberta server. The only unique participant identifier was the participant study number.

Participant names and identifying characteristics were not recorded on the study materials; however, a master form was retained until data capture was complete. All data were entered into the electronic repository as de-identified data following verification and stored on secure servers within the Faculty of Medicine & Dentistry at the University of Alberta.

### Outcomes

**Fig 1:**
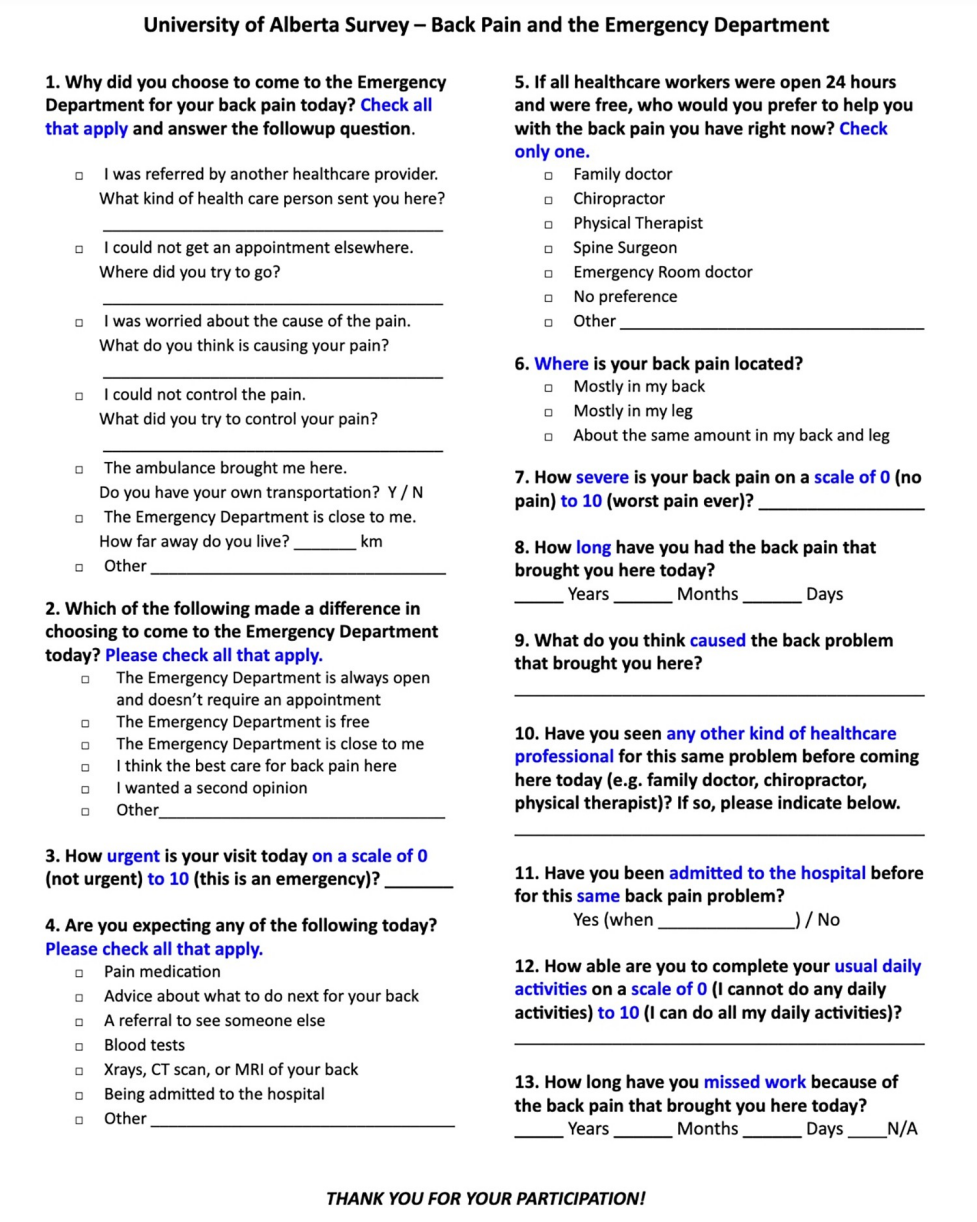
Patient questionnaire.

The study questionnaire was designed to answer our research question within the shortest period of time [11]. Questionnaires required approximately 10 minutes to complete, and included 13 questions (Fig 1). Participants were asked to answer questions relating to their reasons for presentation at the ED (e.g., pain management vs. a lack of alternative options), their expectations, primary care provider visit history, urgency of the ED visit, scale of pain, and preventive health practices prior to the ED visits.

In addition to data collected from the questionnaire, de-identified administrative data for the study period were provided by Data Information and Measurement Reporting (DIMR) within Alberta Health Services (AHS). Additional data contained information on demographics (e.g., age {in years}, sex {male/female}), ED arrival information (e.g., arrival mode, CTAS), ED visit times (e.g., triage, physician initial assessment time {PIA}, consult time, and disposition time), disposition (e.g., admission/discharge; Left without being seen {LWBS}; left against medical advice {LAMA}; death), and post-ED outcomes (e.g., return to ED visits with or without admission within 72 hours).

There was a need to balance research effort and available resources with sample size. For example, enrollment of ~250 participants would provide reasonable precision around mid-range observations (e.g., at 50%, the 95% CI for 250 observations would be ± 6%) as well as low (e.g., at 10%, the 95% CI for 250 observations would be 4%) incidence observations. To obtain 95% CIs approximating ±1% at the mid-range of estimates, enrollment of more than 5000 participants would be needed, which was not feasible.

### Analysis

All study data were exported from REDCap into STATA® (Version 12.0; STATA Corp. LP, College Station, TX) for analysis. Dichotomous results are reported using proportions and comparisons were conducted using Chi-square (χ^2^) statistics. Parametric continuous data are reported as means with standard deviations (SD) and comparisons were completed using unpaired t-test. Nonparametric continuous data are reported as medians with interquartile range (IQR: P75, P25), and comparisons were completed using Wilcoxon rank-sum tests. Factors associated with admission were examined in a logistic regression model using univariate associations at the p <0.2 level as well as common variables from the literature. Variables were entered into the model in a stepwise fashion and deleted when they had a p >0.05 in the combined model. Odds ratios (OR) and 95% CIs are reported for unadjusted and adjusted comparisons. Significance for all tests will be set at p <0.05.

## Results

### Characteristics of study participants

**Fig 2:**
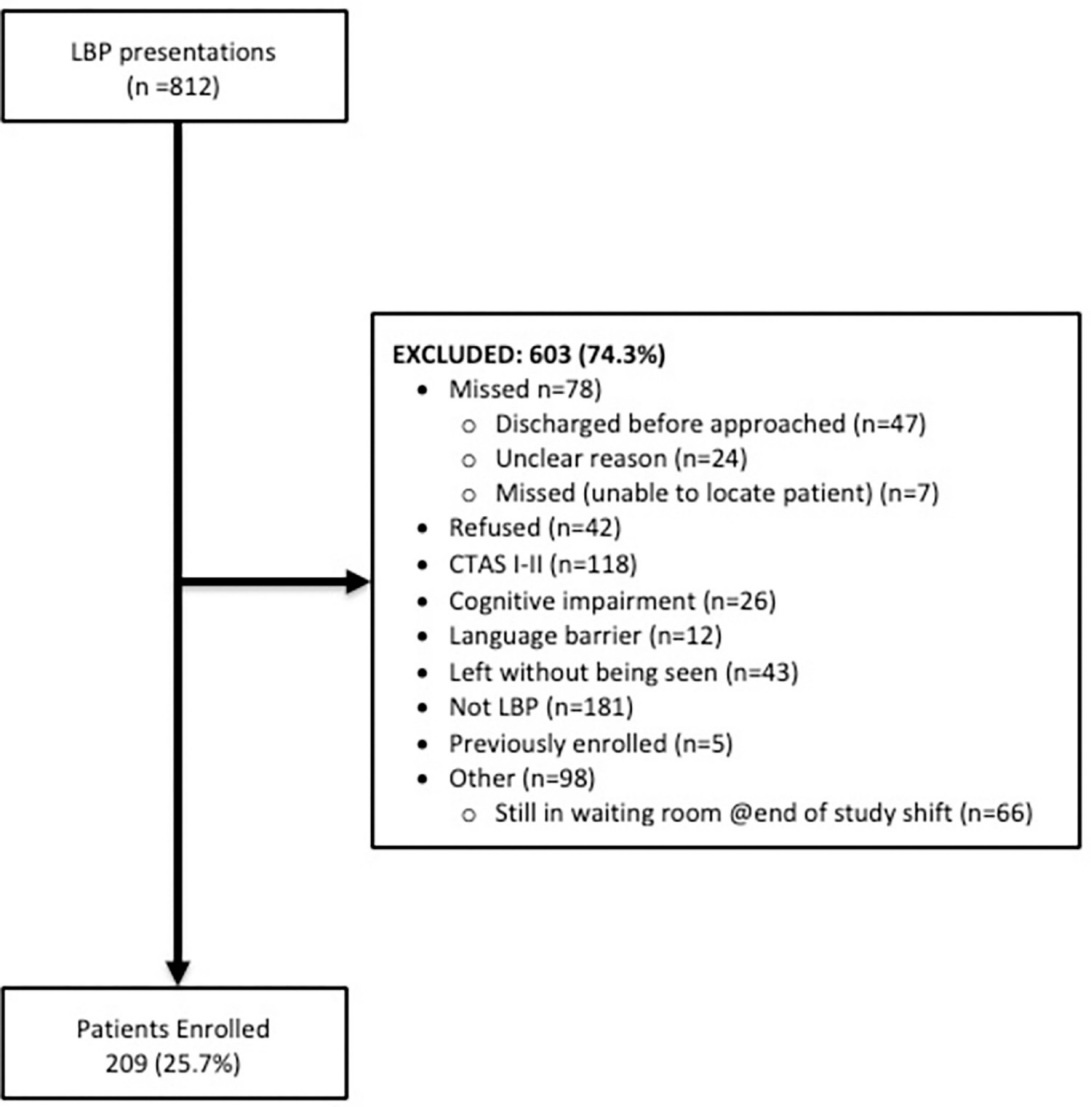
Flowchart of patient enrollment.

Over the study period, 812 persons presented to the UAH ED with a complaint of LBP (Fig 2). These persons were screened against the stated inclusion criteria and 603 were excluded (74.3%) for multiple reasons. Of those excluded, 78 were missed (departed from the ED prior to research assistant arrival), 118 had CTAS scores of 1–2 which required immediate attention and 181 persons completed their ED visit with a non-back pain diagnosis. Additional reasons for non-inclusion were also noted. After these exclusions, 251 participants were eligible to participate of which 209 provided consent to participate in the study (83.3%). The main characteristics and outcomes at ED presentation of the study participants are listed in Table 1.

**Table 1:** Demographic and ED presentation characteristics of patients with acuity low back pain presenting to Canadian emergency department, sub-grouped by disposition status.

Factors	Total (N = 209)	Discharged (N = 186)	Admitted (N = 23)	p-value
Age (years), Median (IQR)	49 (35, 66)	47 (34, 62)	77 (61, 88)	**p<0.0001**
Sex (male; n {%})	105 (50.2)	92 (49.5)	13 (56.5)	0.523
CTAS Score, (n {%})				0.150
CTAS 2/3	156 (74.6)	136 (73.1)	20 (87.0)	
CTAS 4/5	53 (25.4)	50 (26.9)	3 (13.0)	
Arrived by Ambulance, (n {%})	41 (19.6)	27 (14.5)	14 (60.9)	**p<0.0001**
Time of day, (n {%})				0.401
00:01–08:00	40 (19.1)	36 (19.4)	4 (17.4)	
08:01–16:00	149 (71.3)	134 (72.0)	15 (65.2)	
16:01–24:00	20 (9.6)	16 (8.6)	4 (17.4)	
Day of presentation, (n{%})				0.798
Weekdays (M-F)	178 (85.2)	158 (85.0)	20 (87.0)	
Weekend (S-S)	31 (14.8)	28 (15.1)	3 (13.0)	
Investigations (n {%})	79 (38.7)	58 (31.2)	21 (91.3)	**p<0.0001**
HGB	72 (34.5)	51 (27.4)	21 (91.3)	
WBC	71 (34.0)	50 (26.9)	21 (91.3)	
C-creative protein	39 (18.7)	31 (16.7)	8 (34.8)	
Electrolytes	68 (32.5)	49 (26.3)	19 (82.6)	
Urinalysis	41 (19.6)	31 (16.7)	10 (43.5)	
LFTs	31 (14.8)	23 (12.4)	8 (34.8)	
Consultations (n {%})				**p<0.0001**
0	170 (81.3)	170 (100)	0 (0)	
1	31 (14.8)	13 (7.0)	18 (78.3)	
>1	8 (3.8)	3 (1.6)	5 (21.7)	
1^st^ consulted service (n {%})				**0.001**
Orthopedics	10 (27.0)	8 (50.0)	2 (9.5)	
Neurosurgery	3 (8.1)	3 (18.8)	0 (0)	
Other	24 (64.9)	5 (31.3)	19 (90.5)	
ED time to PIA (hrs), Median (IQR)	2.8 (1.4, 4.3)	2.7 (1.4, 4.3)	3.5 (2.2, 4.7)	0.151
ED Length of stay (hrs), Median (IQR)	5.9 (3.8, 9.1)	5.4 (3.5, 7.7)	13.6 (10.5, 22.1)	**p<0.0001**

Note: ED = Emergency Department; CTAS = Canadian Triage and Acuity Scale; HGB = Hemoglobin; LFTs = Liver Function Tests; WBC = White Blood Cell; IQR = Interquartile Range; PIA = Physician Initial Assessment; hrs = hours.

### Main results

Visit characteristics are described in **Table 1** which provides details regarding mode of arrival, triage time, CTAS score, requested procedures and ED LOS. Patients had a median age of 49 (IQR: 35, 66), the male to female ratio was 1:1, and most presented with CTAS 3 severity (153 {73.2%)). Delays to physician initial assessment (median 2.8 hours) and prolonged median length of stay for discharged (5.4 hours) and admitted (13.6 hours) patients indicates an ED suffering from overcrowding. The most common visit outcome was discharge (n = 186, 89%); however, hospitalization (n = 20, 9.6%) and transfer to another facility (n = 3, 1.4%) also occurred.

Responses to the 13-questions (Fig 1) are presented with seven categories including primary reason for attending the ED, secondary reasons for attending the ED, perceived etiology and pain and function (Table 2). Overall, the primary reason for patients choosing the ED was an inability to control pain followed by a need to clarify etiology. The ED was also perceived as a readily available source of care although over half had sought care before coming to the ED. Fewer patients chose the ED because of perceptions about quality of care or cost. In terms of expectations of care, the majority of participants expected to receive pain medication (67.1%) followed by imaging (63.3%) with other interventions being less frequent.

**Table 2:** Responses to a 13-item questionnaire by patients with acuity low back pain presenting to Canadian emergency department, sub-grouped by disposition status.

Questions	Total* (N = 209)	Discharged (N = 186)	Admitted (N = 23)	p-value
**Primary reason for attending ED**				
Unable to control pain	135 (64.6)	120 (64.5)	15 (65.2)	0.947
Concerned about etiology	82 (39.2)	76 (40.9)	6 (26.1)	0.171
Ambulance brought me here	35 (16.7)	23 (12.4)	12 (52.2)	**p<0.0001**
Proximity	24 (11.5)	23 (12.4)	1 (4.4)	0.255
No community clinician available	19 (9.1)	19 (10.2)	0 (0)	0.108
Referred by another practitioner	18 (8.6)	16 (8.6)	2 (8.7)	0.988
Other	57 (27.3)	51 (27.4)	6 (26.1)	0.892
**Secondary Reason for attending the ED**				
ED is always open	93 (44.5)	84 (45.2)	9 (39.1)	0.583
Perceived quality of care	67 (32.1)	55 (30.0)	12 (52.2)	**0.028**
Desired second opinion	32 (15.3)	27 (14.5)	5 (21.7)	0.364
ED is free	8 (3.8)	5 (2.7)	3 (13.0)	**0.015**
Other	79 (37.8)	72 (38.7)	7 (30.4)	0.440
**Perceived etiology**				0.067
Unsure	63 (30.1)	57 (30.7)	6 (26.1)	
Fall	28 (13.4)	22 (11.8)	6 (26.1)	
Bending/Lifting	19 (9.1)	19 (10.2)	0 (0)	
Recurrence of prior back issue	20 (9.6)	20 (10.8)	0 (0)	
Other	79 (37.8)	68 (36.6)	11 (47.8)	
**Pain and function**				
Urgency (10-pt VRS), Median (IQR)^#^	8 (8, 10)	8 (7, 10)	10 (8, 10)	**0.015**
Severity (10-pt VRS), Median (IRQ)^α^	8 (7, 10)	8 (7, 10)	9 (8, 10)	0.213
Duration (days), Median (IRQ)^#^	3 (1, 5)	3 (1, 5)	3 (2, 5)	0.335
Location				0.272
Back only	137 (65.6)	121 (65.8)	16 (72.7)	
Leg only	9 (4.3)	7 (3.8)	2 (9.1)	
Both back and leg	60 (28.7)	56 (30.4)	4 (18.2)	
Missing data	3 (1.4)	2 (1.1)	1 (4.4)	
ADLs (10-pt VRS), Median (IRQ)	3 (0, 5)	3 (0, 5)	0 (0, 3)	**0.004**
Days of work lost	91 (43.5)	87 (46.8)	4 (17.4)	**0.007**
How long (days), Median (IQR)	2 (1, 4)	2 (1, 4)	4 (1, 11)	0.553
**Additional care sought (≥ 1)** ^α^	108 (51.7)	95 (51.1)	13 (56.5)	0.800
Family doctor	83 (76.9)	74 (77.9)	9 (69.2)	0.487
Physical therapist	35 (32.4)	34 (35.8)	1 (7.7)	**0.042**
Chiropractor	33 (30.5)	30 (31.6)	3 (23.1)	0.533
ED physician	18 (16.7)	14 (14.7)	4 (30.8)	0.146
**Expected procedures**				
Pain medication	139 (67.1)	120 (64.5)	19 (82.6)	0.083
Advice	115 (55.6)	103 (55.4)	12 (52.2)	0.771
A referral to another professional	47 (22.7)	41 (22.0)	6 (26.1)	0.661
Blood tests	31 (15.0)	24 (12.9)	7 (30.4)	**0.026**
Imaging	131 (63.3)	116 (62.4)	15 (65.2)	0.790
Admission	25 (12.1)	15 (8.1)	10 (43.5)	**p<0.0001**
Other	33 (15.9)	30 (16.1)	3 (13.0)	0.702
**Preferred clinician if available**				0.727
ED physician	89 (42.6)	76 (40.9)	13 (56.5)	
Family doctor	41 (19.6)	37 (19.9)	4 (17.4)	
Spine surgeon	33 (15.8)	31 (16.7)	2 (8.7)	
No preference	21 (10.0)	19 (10.2)	2 (8.7)	
Physical therapist	8 (3.8)	8 (4.3)	0 (0)	
Chiropractor	3 (1.4)	3 (1.6)	0 (0)	
Other	15 (6.7)	12 (6.5)	2 (8.7)	

*Except where indicated otherwise

^#^ missing = 3

^α^ missing = 2.

Note: ADLs = Activities of Daily Living; ED = Emergency Department; IQR = Interquartile Range; Pt = point; VRS = Verbal Rating Scale.

### Admissions

Factors associated with admission to hospital on univariate analyses were explored using logistic regression. Biological sex was not associated with admission (OR = 1.51; 95% CI: 0.54, 4.21); however, it was considered clinically important and was therefore retained in the final model to correct possible confounding. Overall, after adjustment, only advanced age (OR = 1.05/1 year of increasing age; 95% CI: 1.02, 1.08) and arrival by ambulance (OR = 4.95; 95% CI: 1.79, 13.7) were significantly associated with admission.

## Discussion

Low back pain is an exceedingly common health compliant and when severe, may precipitate a presentation to health care settings, including the ED. This study collected data from consecutive patients presenting to a high-volume, high acuity hospital ED in Western Canada to examine reasons and expectations for presenting to the ED with a complaint of LBP. While numerous studies have evaluated the demographics and visit characteristics of LBP patients in the ED, it is rare for researchers to directly query motivations for attending an ED for LBP.

Our research question, “Why do people with LBP choose to come to the ED” implies that people have a choice of where to seek care. As such, we excluded people who might not have had this choice. This would potentially include patients arriving with a CTAS score of 1 or 2 who would not have made their own choice to come to the ED (e.g., loss of consciousness). In contrast, we included persons brought to the ED by ambulance. Overall, this patient sample was collected without bias, and represents a typical group of patients with LBP in Canadian EDs.

The percentage admitted to hospital in our study with an initial presentation of LBP was close to 10%, a value similar to admittance rates from other Canadian studies [2, 3]. Our analysis suggests that only ambulance arrival and increasing age were associated with hospitalization in this patient sample. Arguably, we suggest that these patients made the most appropriate decision to attend the ED for their complaint of LBP.

What our demographic results tell us is that our study population is consistent with other studies that have investigated ED patient profiles; the LBP ED population tends to be older and fairly equal in sex distribution [2, 3]. Our study population was also similar to the population included in previous ED-based studies in terms of their back pain characteristics. Specifically, a minority of patients attended the ED for their first ever episode of LBP. Like the general population, most who came to the ED had experienced LBP previously and this complaint had impacted their daily life substantially. The duration of their LBP was similar to that reported in community practices [12]. As for the mechanism of injury, the self-reported complaint onset was not overwhelmingly due to direct trauma, something one might assume in an ED presentation. Taken together, these observations suggest there are similarities in the case mix between the LBP seen in the ED and community practices (e.g., those presenting to the ED for LBP are not exceptional in terms of pathology etc.) [13]. Clinically, our finding that the majority of patients with low back pain come to the ED for pain control may place additional pressure on already overcrowded EDs. Given the evolving emphasis on reducing opioid use, imaging and procedures, the ED may not be the most efficient location for care. Alternative strategies may help overcrowded EDs; however, they need to be formally evaluated.

In addition to pain levels, a second prominent reason that patients attended the ED for LBP was related to uncertainly in pain etiology. Overall, back pain conditions having a definitive etiology make up about 10% of the total number of cases [1]. While distinct pain generators for LBP exist (e.g., protruding disc annulus) [1], identifying them in a given individual is not typically possible and is further complicated by non-specific factors [14]. As such, a promising area in preventing such back pain patients from presenting to the ED would for attending clinicians to educate these patients that obtaining a diagnosis for their condition in the ED is unlikely while mitigating their concerns through reassurance [15] and positive messaging [16].

In this tertiary care ED, patients with LBP received delayed assessment and experience prolonged lengths of stay, even when discharged from the ED. Delays have been associated with poor outcomes [17] and this suggests alternatives for ED-based assessment of patients with LBP are needed. Although pain and uncertainty about the cause of LBP were the primary reasons why persons sought care at the ED in our study, perception of convenience seem to play an important role–despite existing preventive health practices the availability of same day and next day appointments with primary care providers in Canada are some of the worst in the 11 countries involved in the Commonwealth Fund survey [18].

Of course, all of the above reasons for attending the ED for LBP may vary according to individual patient expectations. Many participants were expecting medication (most likely those with acute pain) while many were expecting imaging–something that is overused in back pain and especially so in the ED [7, 8, 19, 20].

### Limitations

The main strength of this study was its prospective nature. While we consecutively screened ED presenters over the course of the study period, we were not able to provide research coverage on nights and weekends. This is common in ED-based studies and does not invalidate the results reported. While more severe cases may present at night and perhaps on weekends, this sample includes some patients that arrived overnight/weekends and better represents the populating that had the opportunity to seek alternative care elsewhere. In addition, we excluded patients with extreme pain; however, while we were seeking patients with low acuity LBP, the median pain scores were high for both admitted and discharged patients (9 vs 8; p = 0.213). This study was the first to use a 13-item questionnaire whose psychometric properties have yet to be fully validated. Although different cities, jurisdictions, countries and economic factors likely play a role in shaping who presents to the ED for LBP, it may be possible to generalize these results across countries like Canada where universal health care exists.

## Conclusion

Overall, our data suggest that most patients with LBP present to the ED when their pain is severe and they are seeking pain control and/or they have diagnostic uncertainty. Overall, most patients and providers would agree that the ED is not an ideal location to clarify the diagnosis of chronic conditions; however, without addressing underlying issues related to patient choosing the ED for LBP, issues with timely ambulatory care, lack of guidelines and other factors, the rising trend of ED presentations will not likely change. With these results, future studies should focus on intersectionality issues (e.g., socio- economic status, marital status, race, sex/gender, disability status, etc.) and how they may affect the decisions of these patients.
